# Effects of L-DOPA on Gene Expression in the Frontal Cortex of Rats with Unilateral Lesions of Midbrain Dopaminergic Neurons

**DOI:** 10.1523/ENEURO.0234-20.2020

**Published:** 2021-01-15

**Authors:** Anna Radlicka, Kinga Kamińska, Malgorzata Borczyk, Marcin Piechota, Michał Korostyński, Joanna Pera, Elżbieta Lorenc-Koci, Jan Rodriguez Parkitna

**Affiliations:** 1 Department of Molecular Neuropharmacology, Maj Institute of Pharmacology, Polish Academy of Sciences, Kraków 31-343, Poland; 2 Department of Neuro-Psychopharmacology, Maj Institute of Pharmacology, Polish Academy of Sciences, Kraków 31-343, Poland; 3 Department of Neurology, Faculty of Medicine, Jagiellonian University Medical College, Kraków 31-503, Poland

**Keywords:** frontal cortex, gene expression, L-DOPA, Parkinson’s disease

## Abstract

The development of Parkinson’s disease (PD) causes dysfunction of the frontal cortex, which contributes to the hallmark motor symptoms and is regarded as one of the primary causes of the affective and cognitive impairments observed in PD. Treatment with L-3,4-dihydroxyphenylalanine (L-DOPA) alleviates motor symptoms but has mixed efficacy in restoring normal cognitive functions, which is further complicated by the psychoactive effects of the drug. We investigated how L-DOPA affects gene expression in the frontal cortex in an animal model of unilateral PD. We performed RNA sequencing (RNA-Seq) analysis of gene expression in the frontal cortex of rats with 6-hydroxydopamine (6-OHDA)-induced unilateral dopaminergic lesions treated with L-DOPA, for two weeks. The analysis of variance identified 48 genes with a significantly altered transcript abundance after L-DOPA treatment. We also performed a weighted gene coexpression network analysis (WGCNA), which resulted in the detection of five modules consisting of genes with similar expression patterns. The analyses led to three primary observations. First, the changes in gene expression induced by L-DOPA were bilateral, although only one hemisphere was lesioned. Second, the changes were not restricted to neurons but also appeared to affect immune or endothelial cells. Finally, comparisons with databases of drug-induced gene expression signatures revealed multiple nonspecific effects, indicating that a part of the observed response is a common pattern activated by multiple types of drugs in different target tissues. Taken together, our results identify cellular mechanisms in the frontal cortex that are involved in the response to L-DOPA treatment.

## Significance Statement

The development of Parkinson’s disease (PD) causes dysfunction of the frontal cortex, which contributes to the motor and cognitive impairments observed in PD. L-3,4-dihydroxyphenylalanine (L-DOPA) improves motor symptoms but has mixed efficacy in restoring normal cognitive functions. We investigated how L-DOPA affects gene expression in the frontal cortex in an animal model of unilateral PD. We identified 48 genes with L-DOPA-altered expression levels and gene clusters that follow similar drug-evoked expression patterns. Our findings suggest that the response to L-DOPA was bilateral, involved distinct cell types and overlapped with expression changes evoked by drugs of multiple classes in different tissues. Overall, our results identify cellular mechanisms in the frontal cortex that are involved in the response to L-DOPA treatment.

## Introduction

Parkinson’s disease (PD) is a neurodegenerative disorder that leads to a progressive loss of dopaminergic neurons of the substantia nigra in the ventral midbrain (Hornykiewicz, 1998). The primary symptoms of PD are motor impairments, including tremors, muscle rigidity, and slowness of movement. However, the progression of the disease is also associated with the development of nonmotor symptoms, including cognitive deficits and affective disorders (Martinez-Martin et al., 2007; Aarsland et al., 2010; Riedel et al., 2010). The cognitive symptoms of PD are attributed to impaired function of the frontal cortex (Sawada et al., 2012; O'Callaghan et al., 2014; Mihaescu et al., 2019), possibly resulting from depletion of monoamine neurotransmitters and acetylcholine (Riekkinen et al., 1998; Mattila et al., 2001; Halliday et al., 2014; Buddhala et al., 2015) as well as neurodegeneration affecting the cortex in the final stages of the disease (Braak et al., 2003; González-Redondo et al., 2014; Armstrong, 2017). The impaired function of the frontal cortex also contributes to the motor symptoms of PD because of altered activity and the loss of specificity of efferent neurons (Rowe et al., 2002; Vercruysse et al., 2014). The cellular mechanisms associated with impaired frontal cortex activity remain only partly understood. A number of studies have assessed changes in the transcriptome and proteome in prefrontal cortex samples derived postmortem from PD patients, and in some cases, the results are supplemented with large-scale analysis of single nucleotide polymorphisms (Duke et al., 2006; Dumitriu et al., 2012, 2016; Riley et al., 2014; Hoss et al., 2016). Extensive changes in either transcription or protein abundance were observed, including differences in the expression of genes encoding metallothioneins, the transcription factor *FOXO1* and its network of regulated genes as well as mitochondrial genes, proteasome components, and multiple short RNAs. Nevertheless, the reported results are not in clear consensus with regard to the underlying mechanisms. Additionally, most of the analyses were based on samples from patients suffering from late stages of the disease who had received extensive treatment. Thus, it is difficult to distinguish whether the observed effects should be attributed to late-stage neurodegeneration, neurotransmitter depletion or the effects of long-term medication.

The primary treatment of PD relies on L-3,4-dihydroxyphenylalanine (L-DOPA), a dopamine precursor. L-DOPA is effective in alleviating motor impairments, especially in the initial period of treatment, but displays mixed efficacy against the nonmotor symptoms of PD. For example, L-DOPA was observed to improve working memory (Simioni et al., 2017) and cognitive flexibility but simultaneously increased impulsivity (Cools et al., 2003) and failed to restore impaired sequence learning (Ghilardi et al., 2007) in PD patients. Moreover, the administration of L-DOPA altered frontal cortex connectivity in healthy volunteers and had psychoactive effects, increasing impulsivity and altering performance in tasks dependent on executive functions (Kelly et al., 2009; Shiner et al., 2015). The molecular adaptations involved in the effects of L-DOPA on the frontal cortex remain elusive.

A model commonly used to study the effects of dopamine depletion on brain physiology involves 6-hydroxydopamine (6-OHDA)-induced lesions of dopaminergic neurons (Ungerstedt, 1968; Schwarting and Huston, 1996; Simola et al., 2007). Lesioning is typically performed unilaterally, which permits comparison of the functioning of a dopamine-depleted hemisphere and an intact hemisphere. In addition, 6-OHDA lesioning is widely used because it reproduces a PD-like phenotype resulting from damage to the ventral midbrain neurons and involves the disruption of mitochondrial activities that mediate cytotoxic processes (Simola et al., 2007; Bezard et al., 2013; Kreiner, 2015); thus, 6-OHDA lesioning has potential construct validity as a model of human PD (Hauser and Hastings, 2013). Several studies have examined the effects of 6-OHDA lesions, in some cases followed by L-DOPA treatment, on gene expression in the forebrain and striatum in particular (Berke et al., 1998; Konradi et al., 2004; Heiman et al., 2014; Smith et al., 2016). These studies revealed that neurons on the lesioned side of the striatum exhibited changes in the expression of immediate early-response genes (IEGs), *Grin1* (an essential NMDA receptor subunit), *Stx6* (involved in exocytosis), or *Ldhb* (metabolism). To the best of our knowledge, no studies have comprehensively examined transcriptomic changes evoked by L-DOPA in the frontal cortex.

Here, we investigated the effects of L-DOPA on gene expression in the frontal cortex of rats with unilateral 6-OHDA lesions of the ascending dopaminergic pathways in the medial forebrain bundle (MFB). We analyzed L-DOPA-regulated gene expression in the contexts of potential regulatory mechanisms, cell-type specificity, and similarities to the transcriptional signatures of other drugs.

## Materials and Methods

### Animals

The tissue samples used in this study were prepared as part of a separate study (Lorenc-Koci E and Kaminska K, unpublished observations). All animal procedures were performed in accordance with the European Union guidelines for the care and use of laboratory animals (Directive 2010/63/EU) and were approved by the II Local Institutional Animal Care and Use Committee in Krakow (permit no. 846/2011). Adult male Wistar Han rats (Charles River) with an initial body weight of 300 ± 20 g were maintained in the institutional animal facility (five animals per cage) under standard laboratory conditions (22°C, 12/12 h light/dark cycle, lights on beginning at 7 A.M.). Animals had unlimited access to food and water. The rats were unilaterally lesioned by the infusion of 6-OHDA hydrochloride into the left MFB. The animals were anesthetized with a 1:1 v/v mixture of ketamine (50 mg/kg, Biowet) and diazepam (2.5 mg/kg, Polfa), administered in a volume of 1 ml/kg body weight. A stainless steel needle (0.28-mm outer diameter) was inserted through a hole in the skull, and its tip was placed in the left MFB at the following coordinates, according to the atlas of Paxinos and Watson (1986): A/P = −2.8 mm, L = +1.8 mm, D/V = −8.6 mm. 6-OHDA hydrochloride (8 μg, free base) was dissolved in 4 μl sterile 0.9% NaCl with 0.05% ascorbic acid. The 6-OHDA solution was infused into the MFB through a 10-μl Hamilton syringe at a flow rate of 0.5 μl/min, and the cannula was left in place for another 5 min after the infusion stopped. The animals received desipramine hydrochloride (25 mg/kg, i.p.) 30 min before surgery to prevent noradrenergic neuron damage. After surgery, the rats were allowed to recuperate for two weeks, and then the extent of the lesion was verified using the apomorphine-induced rotation test. Individual animals were placed in automated rotameters (Panlab) after the injection of apomorphine (0.25 mg/kg, s.c.). Following an acclimatization period of 5 min, rats’ movements in both contraversive (right) and ipsiversive (left) directions were recorded. The inclusion criterion for additional experiments was ≥98 contraversive full-body turns in 1 h; however, in the case of samples used in RNA sequencing (RNA-Seq) analyses, the lowest number of rotations was 121.5 (see Extended Data Fig. 1-1). On the day following the rotation test, rats started a 14-d treatment period with L-DOPA (12.5 mg/kg, i.p., once daily) combined with benserazide hydrochloride (6.25 mg/kg, i.p., 30 min before L-DOPA). The control animals received a 0.9% (w/v) NaCl solution. Rats were decapitated 1 h after receiving the last dose of L-DOPA or saline.

**Figure 1.**
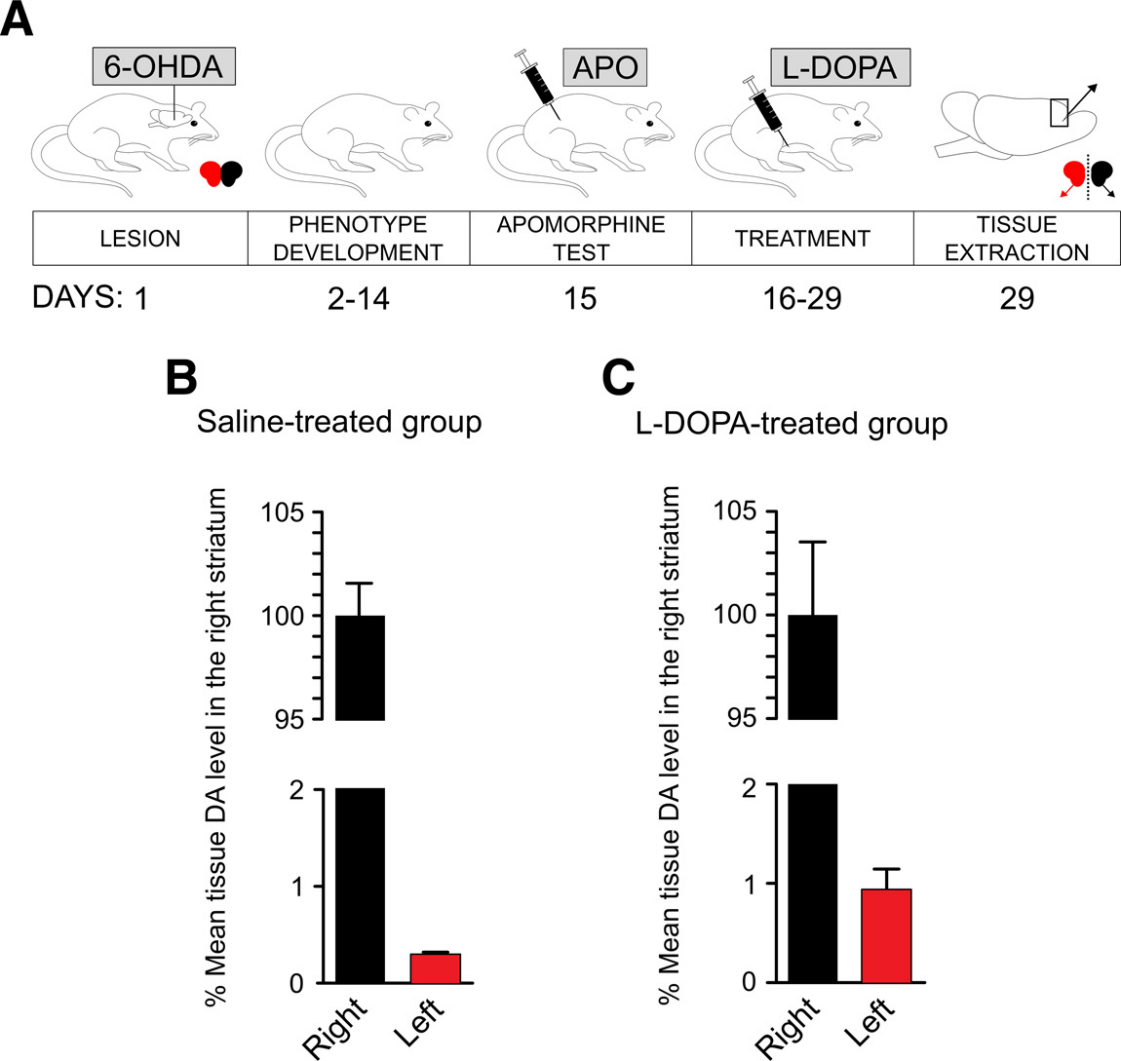
***A***, Treatment scheme of rats with unilateral lesions of dopaminergic neurons as a model of PD. Male adult rats were infused with 8 μg/4 μl 6-OHDA into their left MFB. The toxin was used to kill dopaminergic neurons to produce an animal model of PD. Two weeks later, the animals were injected subcutaneously with 0.25 mg/kg apomorphine (APO) to induce rotational behavior. The number of rotations in 1 h was scored, and only the animals that exhibited at least 98 contraversive rotations (as an indication of sufficient nigral degeneration; for detailed information on number of rotations, see Extended Data Figure 1-1) were used in the following procedures. One day after the APO test, the treatment regime started. Control rats received intraperitoneal saline, whereas the experimental group received intraperitoneal benserazide (6.25 mg/kg) once daily for 14 d followed by L-DOPA (12.5 mg/kg). The animals were decapitated 1 h after the last dose of saline or L-DOPA, and their frontal cortex and striatum tissue was individually dissected from each hemisphere. ***B***, Dopamine (DA) content in the striatum of saline-treated (***C***) and L-DOPA-treated animals is presented as % of the mean of the samples from the right hemisphere.

10.1523/ENEURO.0234-20.2020.f1-1 Extended Data Figure 1-1 Number of contraversive (rightwise) and ipsiversive (leftwise) rotations recorded for 1 h in 10-min intervals in rats from both treatment groups, RINs for frontal cortex samples and information on whether the animals were included in the RNA-Seq study. The rats were tested 14 d after administration of 6-OHDA into the left MFB after injection of apomoprhine (0.25 mg/kg/2 ml, s.c.). N/A, not measured. Download Figure 1-1, XLSX file.

### Measurement of striatal dopamine content

Tissue dopamine content was assessed in the striatal samples separately for the lesioned and nonlesioned sides by reverse-side high-performance liquid chromatography (HPLC) with coulometric detection. Striatal samples were dissected on an ice-chilled plate and then stored at −80°C before being processed. Samples were homogenized in ice-cold 0.1 m perchloric acid with 0.05 mm ascorbic acid and centrifuged (10,000 × *g*, 10 min). The supernatants were filtered through 0.2-μm cellulose filters (Alltech Associates Inc) and injected into the HPLC system (P680 pump, ASI-100 autosampler, TCC-100 thermostated column compartment, Dionex) equipped with a C18 reverse-phase column (150 × 3 mm i.d., 3-μm particle size) fitted with a 10 × 3 mm precolumn (Thermo Fisher Scientific). Detection was conducted using a Coulochem III detector (ESA) equipped with a guard cell (ESA 5020) with the electrode set at 600 mV and a dual electrode analytical cell (ESA 5010). Potentials were set at 350 mV for the first electrode and −220 nV for the second electrode. Temperatures of the column and the analytical cell were maintained at 30°C. The mobile phase consisted of 35 mm citrate/47 mm disodium phosphate buffer (pH 4.2), supplemented with 0.25 mm ethylenediaminetetraacetic acid, 0.25 mm sodium octyl sulfonate, 2.4%methanol, and 1.3% acetonitrile. The measurement was performed at a maintained flow rate of 0.8 ml/min. Dopamine was quantified by peak area comparisons with freshly prepared standards. Chromeleon Chromatography Data System (Thermo Scientific) v6.8 software was used to analyze the collected data.

### RNA-Seq

Frontal cortex samples from both hemispheres were dissected separately on an ice-chilled plate using the olfactory bulbs and the most anterior bifurcation of the middle cerebral artery as orientation points to make cuts in the frontal plane. Striatal tissue was removed from the sections, and the cortices were separated into the left and right sides and stored at −80°C before proceeding. According to the rat brain atlas (Paxinos and Watson, 1986), the following cortical areas were included in the samples: primary and secondary motor, prelimbic, infralimbic, cingulate, insular, orbital, piriform, dorsal peduncular, and primary somatosensory (A/P: 4.2–2.2 mm). Tissue samples were homogenized using a TissueLyser (QIAGEN), and total RNA was extracted with an RNeasy Mini kit (QIAGEN) using a QIAcube (QIAGEN) in accordance with the manufacturer’s protocol. The integrity and concentration of the extracted RNA were assessed using an Agilent RNA 6000 Nano kit on a 2100 Bioanalyzer (Agilent). Based on RNA integrity number values (RIN > 7.2; see Extended Data Fig. 1-1), samples from five animals per group were chosen for RNA-Seq. RNA-Seq was performed as an external service by Novogene. Briefly, poly(A) RNA was isolated from the total RNA samples by the addition of oligo(dT) beads. cDNA libraries were prepared on a template of randomly fragmented mRNA using the NEBNext Ultra II Directional RNA Library Prep kit for Illumina (New England Biolabs). Given that eight samples out of 20 initially contained <200 ng of total RNA, all the samples were processed to prepare cDNA from poly(A) RNA following the low-input protocol. Complete cDNA libraries consisting of reads 250–300 bp in length acquired from the samples were subjected to sequencing on an Illumina system (150 bp, paired-end, 20 million reads per sample).

### Data preprocessing and differential expression analysis

A quality check of the raw RNA-Seq data were performed with FastQC v0.11.8 in R 3.4. The reads were aligned to the Rnor6.0 rat reference genome from the Ensembl database using HISAT2 v2.1.0 (Kim et al., 2019). Transcript counts were normalized to fragments per kilobase of transcript per million fragments mapped (FPKM) values with the Cufflinks v2.2.1 package (Trapnell et al., 2010). We used mixed model two-way ANOVA with lesion as a within-subject factor and treatment as a between-subject factor on log_2_(1 + FPKM) values for each gene to detect statistically significant differences. The Benjamini–Hochberg false discovery rate (FDR) correction for multiple comparisons was used to adjust *p* values. Statistical significance testing was performed on transcripts with mean log_2_(1 + FPKM) values >1. The BioMart interface to the Ensembl database was used for annotation of the transcripts. Hierarchical clustering was performed using distances calculated as (1- Pearson’s *R*^2^). Genes with FDR values <0.05 for the L-DOPA treatment effect are referred to as “differentially expressed” genes. For these genes, we also analyzed exon expression to differentiate between different annotated gene transcripts. Mixed model two-way ANOVA with FDR correction was used to determine the effects of hemisphere, treatment and their interaction on transcript isoform abundance. Mean coverage (more than two reads per site) of alternative splicing variants of two of the differentially expressed genes was plotted using the ggsashimi v0.5.0 package (Garrido-Martín et al., 2018).

### Identification of coexpression networks

Weighted gene coexpression network analysis (WGCNA) was performed using the WGCNA package v1.68 (Langfelder and Horvath, 2008) in R v3.6.1 and applied with a step-by-step approach to construct coexpression networks from genes with mean log_2_(1 + FPKM) values >1. Briefly, a soft-threshold power of 11 was set based on scale-free topology calculations. Then, adjacency values were transformed into a topology overlap matrix (TOM) with TOMtype = “signed,” and TOM-based dissimilarity (dissTOM) was defined as 1-TOM. A network dendrogram of genes was constructed based on average linkage hierarchical clustering and dissTOM with the “dynamic tree cut” method and the minimum module size was set to 30. The resulting gene modules were assigned arbitrary color names for easier reference, and the “gray” module comprised of genes that did not match the inclusion criteria for any other module. Module eigengene values were clustered based on their correlation, and modules identified by cutreeDynamic were merged based on the threshold MEDissThres = 0.5 using the mergeCloseModules function.

### Transcription factor binding

The Seqinspector tool was used to assess overrepresentation of transcription factor-binding sites in the promoter regions of differentially expressed genes (Piechota et al., 2016). Seqinspector uses datasets from ChIP-seq experiments performed in mouse or human cells; thus, we translated the list of rat genes to their mouse homologs and used the mm10 mouse genome assembly as background. Rat genes in the lists were translated to mouse homologs with biomaRt v2.40.5 (Durinck et al., 2005). The following rat genes were excluded because of the existence of more than one or no mouse homologs or differences in the symbols’ names: *Il6r* and *Nfkbia* in the case of the differentially expressed genes, and *Nfkbia*, *Nat8f3*, *Gpr52*, *RGD1311899*, *Rcor2l1*, *Spag5*, *LOC100912481*, and *LOC100911313* in the case of the “salmon” module genes. The resulting records from corresponding sequencing experiments (tracks) were considered statistically significant if their Bonferroni-corrected *p* < 0.05.

### Cell-type specificity of gene expression

Analysis of the brain cell subtypes in which transcripts of the differentially expressed genes were present was performed using RNA-Seq Data Navigator from the Cell Types Database (Allen Institute for Brain Science, 2015). The database is based on RNA-Seq profiling of mouse cortical cells, and we used the list of murine homologs of the rat genes indicated by ANOVA as described above. *Dipk2a*, *Noct*, and *Cavin2* mouse genes were not recognized by the database and were excluded from the analysis along with *Nfkbia* and *Il6r*. The result of this analysis was the prevalence of transcripts of our differentially expressed genes within different cell subtypes (Tasic et al., 2018). The exported group fraction values for each gene were then used to plot a heatmap.

### Annotation enrichment analysis

The differentially expressed and salmon cluster genes were analyzed for annotation enrichment using the enrichR package v2.1, an R interface to the Enrichr web server (Chen et al., 2013; Kuleshov et al., 2016). Enrichr uses the human genome as the reference set; thus, we used biomaRt v2.40.5 to identify human homologs of rat genes. Genes with different symbols, genes without a human homolog, and genes that had >1 human homolog were excluded from the analysis. The following genes were excluded: *Zfp189* and *Nfkbia* from the list of differentially expressed genes and *RGD1311899*, *Zfp189*, *Nat8f3*, *Nfkbia*, *Gpr52*, *Sik1*, *Zfp521*, *Rcor2l1*, *Spag5*, *LOC100912481*, and *LOC100911313* from the list of genes included in the salmon WGCNA module. The results were considered significant at an adjusted *p* < 0.05.

### Code accessibility

The code described in the paper is freely available online at https://github.com/ippas/ifpan-annaradli-ldopa.

## Results

### L-DOPA-induced gene expression

We used RNA-Seq to assess the effects of L-DOPA treatment on gene expression in the frontal cortex in animals with dopaminergic lesions. Experiments were performed on samples derived from animals that underwent induction of unilateral lesions of dopaminergic neurons with 6-OHDA followed by 14 d of L-DOPA treatment. A diagram summarizing the procedure is shown in Figure 1*A*. The lesions caused >99% loss of dopamine content in the ipsilateral striatum on the lesioned side, and similar efficiency was noted in the L-DOPA-treated group (Fig. 1*B,C*). We dissected the left and right frontal cortices and separately isolated total RNA from each of them. For both treatment groups, i.e., saline or L-DOPA, we analyzed five paired samples (left and right cortices from the same rat, 20 samples in total). RNA-Seq of poly(A)-enriched cDNA yielded on average 26.4 million pairs of raw reads per sample. The sequence reads were mapped to the Ensembl rat genome assembly Rnor6.0 using HISAT2. The reads were aligned to a total number of 33,883 sequences, and counts were normalized to FPKM values. There were 12,455 genes with a mean log_2_(1 + FPKM) value >1. These genes included 12,034 protein-coding genes, 259 pseudogenes, 98 long intergenic noncoding RNAs, two mitochondrial rRNAs, one nucleolar rRNA, two ribozymes, three scaRNAs, and one snoRNA. Raw RNA-Seq data are available at BioProject (https://www.ncbi.nlm.nih.gov/bioproject/; accession no. PRJNA547879).

**Figure 2.**
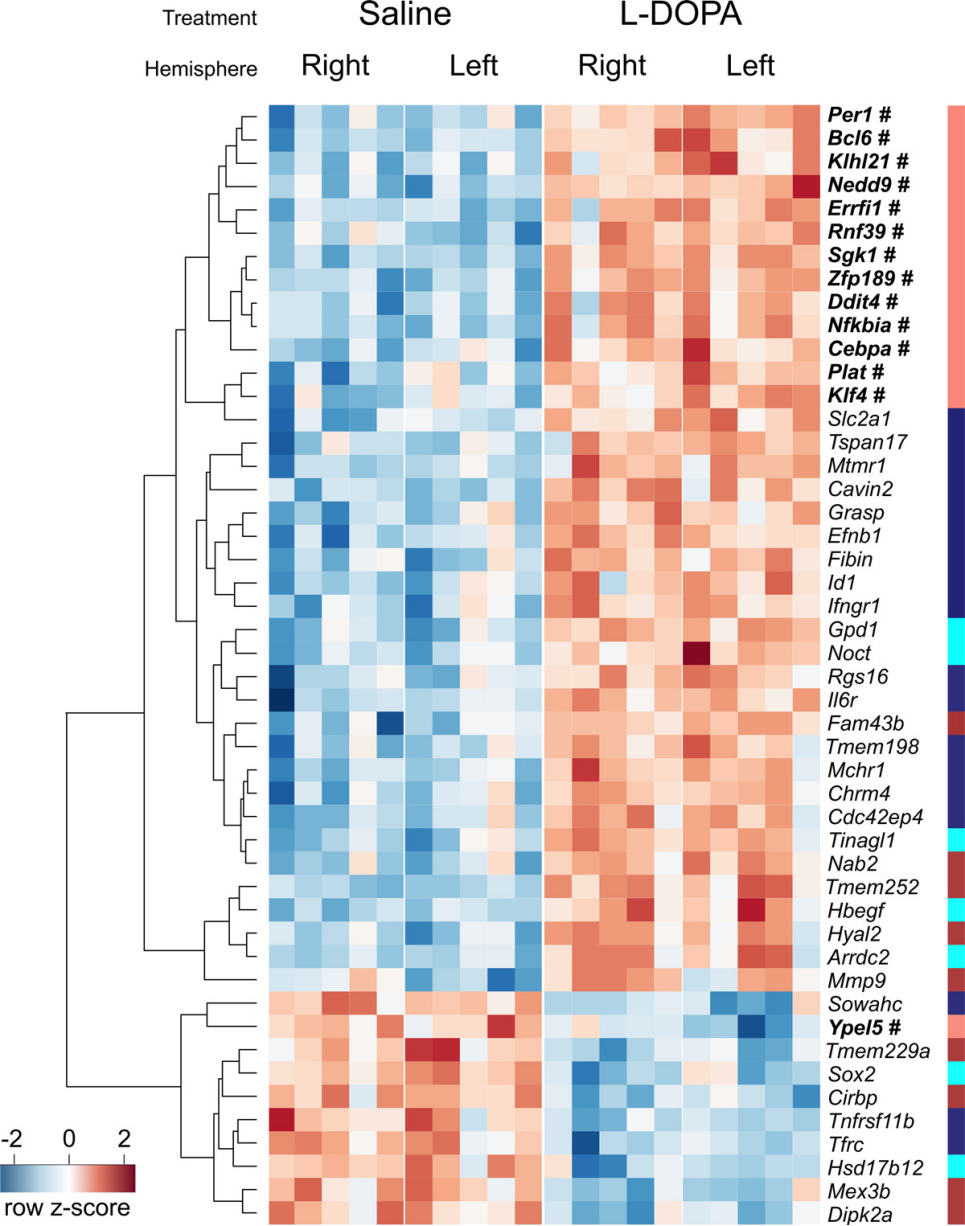
Gene expression changes evoked by L-DOPA in the frontal cortex of rats with unilateral lesions of the dopaminergic system. A total of 48 genes exhibit altered transcript abundance after L-DOPA treatment in the frontal cortex of rats with unilateral 6-OHDA lesions (two-way ANOVA, FDR < 0.05). Each column represents one sample (left or right frontal cortex), and rows correspond to genes as indicated on the right. Colors represent normalized expression levels. Genes are ordered based on hierarchical clustering, and the dendrogram is presented on the left. Additionally, the colored stripe on the right shows the assignment of genes to modules from the WGCNA. Genes belonging to the salmon module are highlighted in bold and marked with a #. For detailed data on the differentially expressed genes, see Extended Data Figure 2-1. For detailed data on alternative transcript isoforms of the differentially expressed genes, see Extended Data Figures 2-2, 2-3. Additionally, the results of overrepresentation analyses are presented in Extended Data Figures 2-4 (transcription factor-binding sites) and 2-5 (drug-induced expression signatures).

To identify differentially expressed genes, we performed mixed model two-way ANOVA with FDR correction for multiple comparisons. The comparison of the lesioned versus nonlesioned side was treated as a “within” factor to limit potential effects of differences in the extent of the lesion between animals. Out of 12,455 genes, there were 48 genes with FDR values smaller than 0.05 for the “treatment” factor, as summarized in Figure 2. No genes were differentially expressed between the lesioned (left) and nonlesioned (right) frontal cortices, and no statistically significant interactions between effects were observed (Extended Data Fig. 2-1). L-DOPA treatment caused an increase in the abundance of transcripts corresponding to 38 genes (upper part of the heatmap) and downregulated 10 genes (lower part). All the differentially expressed genes were protein-coding genes and had functions related to the immune response (*Bcl6*, *Ifngr1*, and *Il6r*), modification of the extracellular matrix (*Hyal2* and *Mmp9*), neuronal signal transduction (*Chrm4*), circadian rhythm (*Per1*), cellular uptake (*Tfrc* and *Slc2a1*), stress response (*Sgk1*), cell differentiation (*Sox2* and *Nedd9*), and response to hypoxia (*Ddit4*). Moreover, we noted that several genes, including *Per1*, *Sgk1*, *Errfi1*, *Id1*, and *Klf4*, were associated with the immediate early response. Mean transcript abundancies of the differentially expressed genes normalized as log_2_(1 + FPKM) were in the range of 1.16 (*Mmp9*) to 6.75 (*Tspan17*).

10.1523/ENEURO.0234-20.2020.f2-1 Extended Data Figure 2-1 List of differentially expressed genes with Ensembl IDs, gene symbols, mean log2(FPKM +1) values, FDR values for all factors, fold changes for each contrast, and the WGCNA modules to which the genes were assigned. Download Figure 2-1, XLSX file.

10.1523/ENEURO.0234-20.2020.f2-2 Extended Data Figure 2-2 List of alternative transcripts of the differentially expressed genes with Ensembl transcript IDs and names, gene symbols, log2(FPKM +1) values in each sample and mean for all samples, uncorrected *p* and FDR values for all factors, and fold changes for each contrast. Download Figure 2-2, XLSX file.

**Figure 3.**
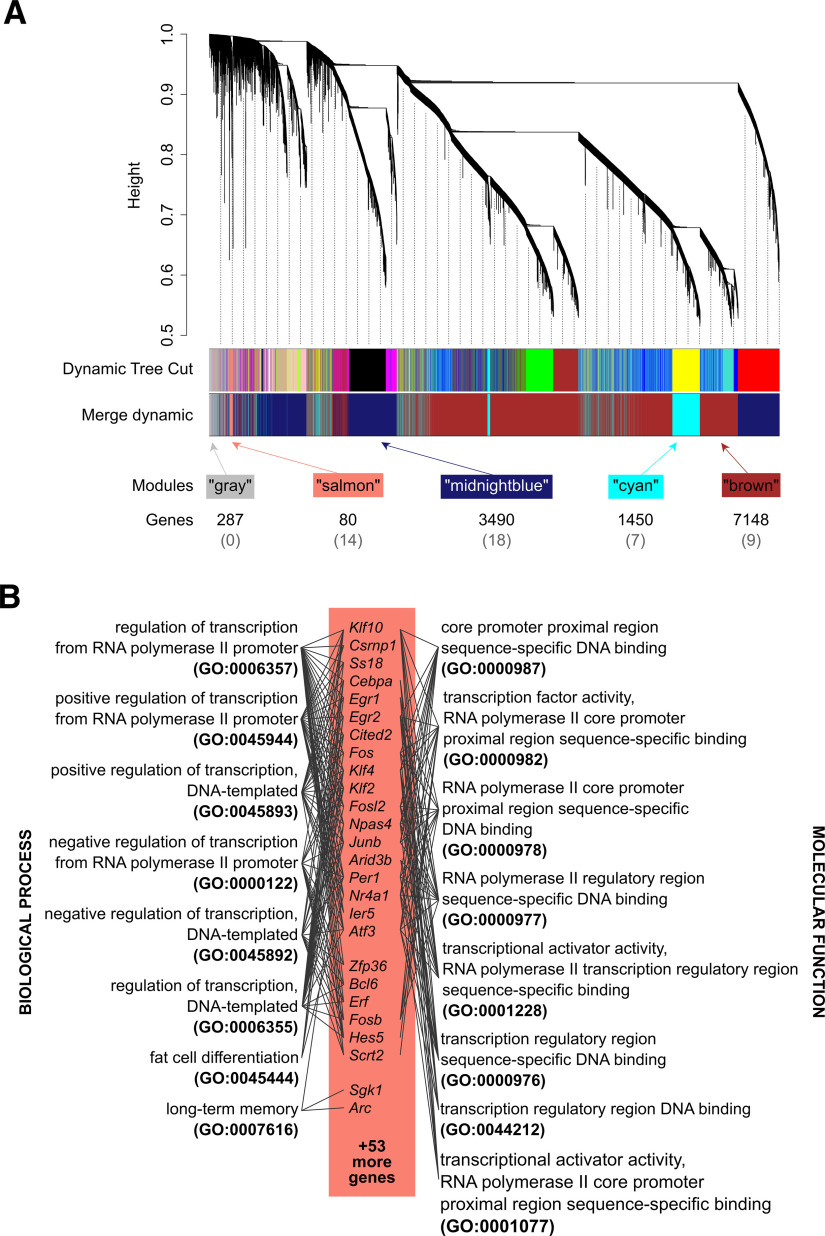
L-DOPA-regulated gene coexpression networks in the rat frontal cortex. ***A***, Graphic representation of gene assignment of the 12,455 genes [with log_2_(FPKM + 1) values >1] to WGCNA modules. Each branch of the dendrogram and colored stripe represent one gene, and the colors indicate assignment to a particular module named by color. The dynamic tree cut method detected 17 modules (the upper block of colored stripes), which were further joined with the “merge dynamic” method at a threshold of 0.5 into five resulting modules (the lower block). The numbers below the blocks indicate the total number of genes in a module, and those in brackets represent the number of genes in the module that overlap with the differentially expressed genes. The module eigengenes for each sample are shown in Extended Data Figure 3-1. ***B***, The salmon module included many immediate early genes and was enriched in GO terms related to the process of transcription. Biological process GO terms are on the left, and molecular function terms are on the right. Gray lines connect the terms with the corresponding genes. For full results of overrepresentation analyses of the salmon module, please refer to Extended Data Figures 3-2 (transcription factor-binding sites), 3-3 (GO), and 3-4 (drug-induced expression signatures).

10.1523/ENEURO.0234-20.2020.f2-3 Extended Data Figure 2-3 Sashimi plots of two of the differentially expressed genes with alternative transcript isoforms: (***A***) *Mmp9* and (***B***) *Sgk1*. The upper parts represent splice junctions and mean coverage for each sample group in accordance with genome coordinates of the aligned isoforms. The coverage is presented only for sites with more than two reads. Asterisk (*) marks transcript isoforms whose abundances were significantly altered by L-DOPA (FDR for treatment < 0.05), whereas the $ indicates the *Mmp9* isoform with significant abundance differences between hemispheres. Download Figure 2-3, TIF file.

**Figure 4.**
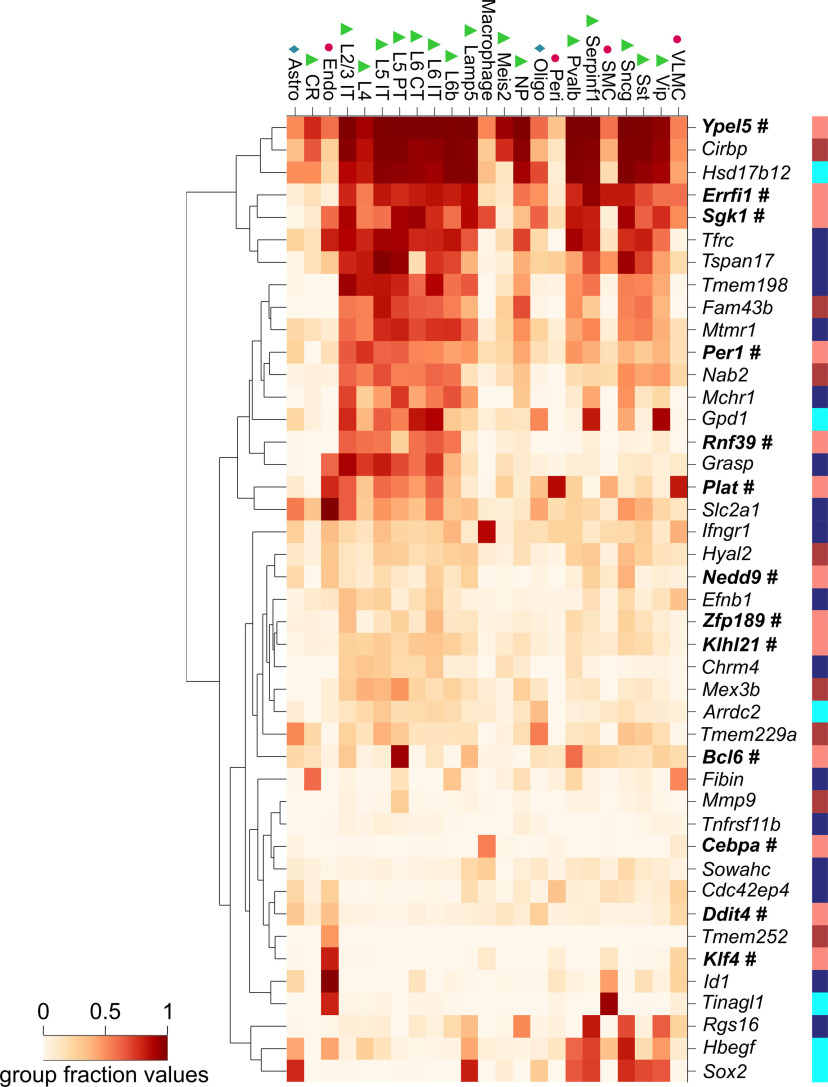
Analysis of cell-type specificity of the expression of genes differentially expressed in the rat frontal cortex after L-DOPA treatment. The heatmap includes genes that passed the differential expression criterion and had mouse homologs. Each column reflects a cell subtype, based on the Allen Atlas classification, and rows correspond to genes. The color of the squares in the heatmap indicates the fraction of the cells belonging to the subtype that was found to express a particular gene (legend shown in the bottom left). Symbols above the heatmap mark broader cell categories: triangles, neurons; diamonds, glia; dots, cells associated with the vascular system. The colored stripe on the right indicates the assignment of genes to modules from the WGCNA. Genes belonging to the salmon module are highlighted in bold and marked with a #. Astro, astrocytes; CR, Cajal–Retzius cells; CT, corticothalamic neurons; Endo, endothelial cells; IT, intratelencephalic neurons; L, cortical layer; NP, near-projecting neurons; Oligo, oligodendrocytes; Peri, pericytes; PT, pyramidal tract neurons; SMC, smooth muscle cells; VLMC, vascular leptomeningeal cells.

10.1523/ENEURO.0234-20.2020.f2-4 Extended Data Figure 2-4 Enrichment of transcription factor-binding sites in the loci of the differentially expressed genes. Download Figure 2-4, XLSX file.

10.1523/ENEURO.0234-20.2020.f2-5 Extended Data Figure 2-5 Significant overlaps between differentially regulated genes and transcriptomic signatures from GEO upregulated and downregulated genes (GEOPertUp, GEOPertDown) and the Drug Signature Database (DSigDB). Download Figure 2-5, XLSX file.

For 13 out of 48 differentially expressed genes, alternative transcript isoforms were identified, namely, *Dipk2a* (2 splicing variants), *Grasp* (2), *Hsd17b12* (2), *Hyal2* (2), *Mmp9* (2), *Mtmr1* (2), *Nedd9* (3), *Rnf39* (3), *Sgk1* (4), *Slc2a1* (2), *Tinagl1* (2), *Tspan17* (2), and *Zfp189* (2; see Extended Data Fig. 2-2). Mixed model two-way ANOVA with FDR correction for multiple comparisons revealed two significant results at the transcript level. L-DOPA altered the transcript abundance of *Dipk2a-202*, *Grasp-201*, *Mmp9-201*, *Nedd9-201*, *Rnf39-201*, *Sgk1-201*, *Sgk1-204*, *Slc2a1-201*, *Tinagl1-201*, *Tspan17-201*, and *Zfp189-201*. The *Mmp9-201* isoform was also differentially expressed on the contralateral side (FDR < 0.05 for the “hemisphere” factor; for details, see Extended Data Fig. 2-3*A*). Additionally, two out of four splicing variants of *Sgk1* were regulated by L-DOPA in the opposite way. Specifically, the *Sgk1-201* isoform abundance increased after treatment, whereas *Sgk1-204* decreased (Extended Data Fig. 2-3*B*).

### Identification of coexpression networks

To identify networks of coregulated genes, we employed WGCNA. This method addresses some of the weaknesses associated with the identification of differentially expressed genes using null hypothesis testing, such as arbitrary significance criteria or the assumption that the expression of individual genes is independent. We performed step-by-step WGCNA with a soft-threshold power of 11, and the lowest number of the scale-free topology *R*^2^ was >0.8. The 12 455 genes were clustered with the dynamic tree cut method, which resulted in the detection of 17 modules, each labeled with a color (color names are arbitrary and only used for easier reference; Fig. 3*A*). Five modules were detected at the merging threshold of 0.5 (Extended Data Fig. 3-1): “brown” with 7148 genes (including 6909 protein-coding genes), “cyan” with 1450 genes (1401), gray with 287 genes (257), “midnight blue” with 3490 genes (3388), and salmon with 80 genes (79). The modules in the figure are not continuous, as it was not possible to accurately represent distances based on eigenvalues in a two-dimensional plot.

10.1523/ENEURO.0234-20.2020.f3-1 Extended Data Figure 3-1 Module eigengenes (MEs) of the five WGCNA modules after merging the modules identified by the dynamic tree cut method. The columns indicate modules, and the rows represent samples. Download Figure 3-1, XLSX file.

10.1523/ENEURO.0234-20.2020.f3-2 Extended Data Figure 3-2 Enrichment of transcription factor-binding sites in the loci of genes assigned to the salmon WGCNA module. Download Figure 3-2, XLSX file.

10.1523/ENEURO.0234-20.2020.f3-3 Extended Data Figure 3-3 Significantly enriched GO terms from the biological process and molecular function branches in the list of genes assigned to the salmon WGCNA module. Download Figure 3-3, XLSX file.

10.1523/ENEURO.0234-20.2020.f3-4 Extended Data Figure 3-4 Significant overlaps between genes in the salmon WGCNA module and transcriptomic signatures from upregulated and downregulated genes in the GEO (GEOPertUp and GEOPertDown, respectively), the Drug Signature Database (DSigDB) and the DrugMatrix Database. Download Figure 3-4, XLSX file.

The smallest of the identified clusters, the salmon cluster, exhibited high overlap with the ANOVA results and included 14 out of the 48 differentially expressed genes (Fig. 2; Extended Data Figs. 2-1, 2-3*A*). Seventy-nine out of 80 genes in this module were protein-coding genes (one was a pseudogene), and the module included several IEGs (e.g., *Fos*, *Fosb*, *Junb*, *Egr1*, *Arc*, and *Npas4*), a large proportion of which were related to transcription processes (Fig. 3*B*). Of the 14 overlapping genes, 13 were clustered in the dendrogram in Figure 2. The proximity of these 13 genes on the heatmap dendrogram is consistent with expectations as clustering is based on an approach similar to that of the identification of coexpressed modules. The overlap in results from the two analytical methods cross validates the effects on L-DOPA on transcripts associated with immediate early gene expression, which was hemisphere independent (no specific effect of the lesion on the ipsilateral side). The other coexpression modules exhibited relatively low overlap with the differentially expressed genes (Fig. 3*A*). The midnight blue module contained 18 differentially expressed genes, 15 of which were upregulated and three of which were downregulated. Of these 15 genes, nine genes (*Slc2a1*, *Tspan17*, *Mtmr1*, *Cavin2*, *Grasp*, *Efnb1*, *Fibin*, *Id1*, and *Ifngr1*) were adjacent in the heatmap (Fig. 2). Additionally, the remaining six upregulated genes were clustered on the dendrogram (branches containing *Tmem198*, *Mchr1*, *Chrm2*, and *Cdc42ep4* and *Rgs16* and *Il6r* individually). However, an overlap of 18 among 3490 is very low. Even smaller overlaps were observed in the case of the brown (nine genes among 7148) and cyan (seven genes among 1450) modules. The gray module, a collection of genes that were not allocated to any module of coexpressed genes, did not include any differentially expressed genes. Therefore, we performed further analyses of gene promoters and annotation enrichment on the salmon module to obtain a result complementary to the set of differentially expressed genes.

### Cell-type specificity of gene expression

L-DOPA impacts cells of different types and subtypes (Yanovsky et al., 2011; Gantz et al., 2015; Masukawa et al., 2017; Sagot et al., 2018); therefore, we analyzed the differentially expressed genes to assess their expression frequency within particular cell subtypes. We used large-scale single-cell RNA-Seq murine datasets from the Allen Institute for Brain Science to identify cell types with previously confirmed transcription of the differentially expressed genes (Allen Institute for Brain Science, 2015; Tasic et al., 2018). For each gene of interest, we queried the fraction of cells of each type in which it had been found to be present (the criterion in the original paper was CPM ≥ 1). The reported cell classification was based on the expression of marker genes and clustering of transcriptomic profiles using WGCNA and principal component analysis. The analysis results are summarized in the heatmap shown in Figure 4. The use of fractions permits the assessment of the ubiquity of gene expression; however, it is not necessarily indicative of high levels of transcription. In general, the heatmap shows that the differentially expressed transcripts vary greatly in the ubiquity of their expression. The upper part clusters transcripts present in most types of cells and essentially all types of neurons. *Ypel5* is an example of a differentially regulated transcript that was highly ubiquitous (fraction of cells > 0.33 across all the cell subtypes). A group of differentially expressed transcripts was prevalent in glutamatergic and GABAergic neurons, i.e., *Errfi1*, *Fam43b*, *Mtmr1*, *Tmem198*, and *Tspan17*. Genes with relatively specific expression restricted to particular subpopulations of GABAergic neurons (i.e., parvalbumin or serpin F positive) included *Hbegf* and *Rgs16*, whereas *Rnf39* expression was detected mainly in glutamatergic neurons. None of the differentially expressed genes were specifically expressed by a single type of neuron. The most specific expression was observed for *Cebpa* and *Klf4*, which appeared restricted to macrophages and endothelial cells, respectively. Some transcripts, such as *Mmp9* and *Tnfrsf11b*, were detected in less than one-third of cells in all subtypes. To indirectly confirm cell-type specificity we also examined the expression of specific gene isoforms. In the cases of *Sgk1-204* and *Sgk1-201*, it was previously reported that induction of the former is specific to neurons, whereas the latter is enriched in both neurons and astrocytes (Slezak et al., 2013).

### Gene promoter and annotation analyses

Next, we compared the lists of differentially expressed genes and those included in the salmon module with available gene annotation sources to explore shared regulatory factors, overlapping involvement in biological processes or functions, and similarities in transcriptional signature to reported effects of other drugs. The promoter regions of genes with differential expression and the salmon module for transcriptional factor binding were analyzed using Seqinspector. The analysis identified eight transcription factors with significantly increased binding in the promoter regions of the differentially regulated genes: early growth response protein 1 (EGR1), EGR2, glucocorticoid receptor (GR), hypoxia-inducible factor 1-α (HIF1A), myoblast determination protein 1 (MYOD), myogenin (MYOG), neuronal PAS domain protein 4 (NPAS4), and transcription factor 3 (TCF3; Extended Data Fig. 2-4). In the case of the salmon module genes, the analysis identified seven transcription factors, namely, MYOG, GR, NPAS4, and serum response factor (SRF), CCAAT/enhancer-binding proteins β and δ (CEBPB and CEBPD, respectively), CREB-binding protein (CBP; the complete list is presented in Extended Data Fig. 3-2). The results are consistent with previous analyses demonstrating that EGR1, EGR2, NPAS4 and SRF are involved in the activation of the expression of IEGs (Herdegen and Leah, 1998; Benito and Barco, 2015). Additionally, GR-dependent expression appears in the acute response to treatment with most drugs, including psychostimulants and drugs acting on dopamine receptors (Piechota et al., 2012; Zygmunt et al., 2019). The results of ontology analysis of the genes assigned to the salmon module with enrichR also appear consistent with the idea of the activation of IEGs. A large proportion of these genes act as transcription regulators, and accordingly, 6 out of 8 enriched Gene Ontology (GO) biological process terms as well as eight out of eight enriched GO molecular function terms were related to transcriptional processes (Fig. 3B; for full results, see Extended Data Fig. 3-3). One of the GO biological process terms with significant overrepresentation was “long-term memory” (GO:0007616), and the term was assigned to *Npas4*, *Arc*, and *Sgk1*. On the other hand, the 46 differentially expressed genes tested were not significantly enriched in GO terms.

Next, we assessed overlaps between L-DOPA-regulated genes and gene sets related to the response to pharmacological treatment in the following databases: the Drug Signatures Database (Yoo et al., 2015) and the Gene Expression Omnibus (GEO) drug perturbations database (full analysis results are shown in Extended Data Fig. 2-5). A large number of significant overlaps were identified, including expression patterns associated with steroids (e.g., dexamethasone, estradiol, and corticosterone), antidiabetics (rosiglitazone and insulin), cytotoxic and cytostatic agents (e.g., vemurafenib and actinomycin D), vitamins (e.g., K3, retinoic acid, and ascorbic acid), drugs targeting immune responses (e.g., lipopolysaccharide and etanercept), toxins (e.g., sodium arsenite, vanadium pentoxide, and benzene), and drugs of abuse (morphine, heroin, cocaine, and ethanol). The results of the analysis of the salmon module genes were largely similar (Extended Data Fig. 3-4) with additional significant results from the current release of the DrugMatrix Database (United States Department of Health and Human Services, 2010) that did not reveal any significant overlap with the list of differentially expressed genes. Importantly, these transcriptional signatures were reported in different organisms and tissues, including the rat liver, a human breast adenocarcinoma cell line and the murine striatum. We noted that 41 of the L-DOPA-regulated genes and 65 of the genes in the salmon module were represented in both of the drug-related expression signature databases (i.e., Drug Signatures Database and GEO database). This finding indicates that a large subset of genes influenced by L-DOPA are also activated by multiple other drugs, which may represent a nonspecific response. For instance, among the 319 significant overlaps between the sets from all the drug signature databases and the 46 differentially expressed genes used in the analysis, *Ddit4* appeared 155 times, and *Hbegf* appeared in 114 cases (Extended Data Fig. 2-5). Similar findings were noted for genes in the salmon module: *Atf3* appeared in 724 out of 1166 total drug-regulated transcription signatures, 594 signatures included *Egr1*, and 421 signatures listed *Dusp1* (Extended Data Fig. 3-4). Conversely, 34 of the differentially expressed genes were present in the enrichment results of all the relevant drug-related databases, and among genes in the salmon module, 19 were present in the sets from the salmon significantly overrepresented databases. The 34 differentially expressed genes in the drug sets were not associated with any of the specific cell subtypes based on the analysis results shown in Figure 4. These results may suggest that genes not included in the overlaps with the transcriptional signatures of other drugs are associated with molecular mechanisms specific to L-DOPA action. The four genes that were not present in the transcriptional signatures included *Grasp*, *Dipk2a*, *Noct*, and *Cavin2.* The latter three were not recognized by the Allen Institute for Brain Science’s Cell Types Database analytical tool. This leaves *Grasp*, which appears to be expressed predominantly in neurons as well as endothelial cells (Fig. 4).

Finally, we performed a direct comparison of the results of gene expression analysis with those of a recent comprehensive analysis of L-DOPA-induced transcription in the rat striatum (Smith et al., 2016). A previous study identified 95 genes with decreased abundance in striatal tissue in a unilateral 6-OHDA rat model of L-DOPA-induced dyskinesia, of which 19 were included in the salmon module (e.g., *Arc*, *Atf3*, *Egr1*, *Egr2*, and *Nr4a1*) and four in the list of the differentially expressed genes (i.e., *Klf4*, *Per1*, *Cdc24ep4*, and *Nab2*). However, it should be noted that there are important methodological differences between the cited study and this report, including the use of a different dose of L-DOPA (4 mg/kg) in the case of the experiments reported by Smith et al. (2016).

## Discussion

We found that L-DOPA treatment in rats with unilateral lesions of dopaminergic neurons largely induced bilateral changes in gene expression in the frontal cortex. The differentially expressed genes are functionally diverse and include products engaged in the immediate early response of the cell. Moreover, we also observed that in the case of some genes, changes in expression exclusively affected selected isoforms. Analysis of the cell-type specificity of gene expression indicated that transcription changes potentially occurred in both neuronal and nonneuronal cell types. Finally, we found that with the few exceptions, the differentially expressed genes overlapped with genes regulated by other, functionally unrelated drugs.

In general, 6-OHDA-induced lesioning of dopamine neurons in rats is a widely used model of PD (Simola et al., 2007). Several studies demonstrated differences in IEG induction following the administration of a D1-like receptor agonist and the contents of dopamine and its metabolites between the lesioned and nonlesioned hemispheres in animals with unilateral 6-OHDA lesions, particularly in the striatum (Perese et al., 1989; Berke et al., 1998; Walker et al., 2013), and at the level of monoamine neurotransmitters in the substantia nigra, hippocampus and frontal cortex (Kamińska et al., 2017). We found no genes with significant differences in expression between the lesioned and intact sides of the frontal cortex, with a single possible exception of an *Mmp9* transcript. Analysis of alternative transcript isoforms of the differentially expressed genes indicated an increased expression level of the longer *Mmp9* splicing variant on the contralateral (nonlesioned) side. Nevertheless, the almost universal bilateral changes in expression indicate that either the unilateral lesion had no appreciable basal effect on homeostasis in the frontal cortex, or the effects on both sides were the same. We would argue that the former is more likely based on the following reasons. First, previous results confirmed depletion of dopamine on the lesioned side of the frontal cortex of rats that underwent the same treatment (Kamińska et al., 2017). Second, the expression of markers of glial activation (e.g., *Gfap* and *Aif1*) appeared normal and not indicative of glial proliferation. This finding would imply a major difference from the observed sensitization of dopamine receptor D1 signaling observed in the dopamine-depleted striatum (Berke et al., 1998). Although the bilateral effects in the frontal cortex may appear counterintuitive, it was reported that a unilateral lesion of the nigrostriatal pathway was associated with disrupted metabolic connectivity between the ipsilateral auditory cortex and both frontal cortices, whereas regional glucose metabolism was significantly correlated between the lesioned striatum and both motor cortices (Im et al., 2016). Similarly, in hemiparkinsonian rats, increases in the mRNA abundance of *Arc* in the primary motor cortex following acute or chronic L-DOPA treatment were bilateral despite a unilateral depletion of TH-positive nerve fibers (Lindenbach et al., 2015). The effects on *Fos* were lateralized (Lindenbach et al., 2015); however, another report based on a similar lesion model showed bilateral increases in *Fos* after L-DOPA treatment (Monnot et al., 2017). Additionally, it should be noted that D1 receptor sensitization in the lesioned striatum was modulated by inputs from the contralateral prefrontal cortex (Herve et al., 1989). The transcriptomic response to L-DOPA in the striatum and frontal cortex in rodents with unilateral lesions of the nigrostriatal pathway is thus spatially distinct and may follow tissue-specific regulatory mechanisms. Finally, we may not exclude the possibility that unilateral changes restricted to one of the substructures (e.g., primary or secondary motor cortex) included in the samples we analyzed, would be diluted and thus missed in our analysis. We find this unlikely however as the studies that assessed effects of dopamine receptor agonists or L-DOPA on expression of *Fos* or other immediate-early genes exhibit broad activation of transcription across all cortices (Berke et al., 1998; Monnot et al., 2017).

A comparison of the effects of L-DOPA on gene expression in the frontal cortex with results reported in the striatum (Smith et al., 2016) reveals some overlap in the differentially regulated genes identified. However, the changes in transcription appear opposite in the case of several putative IEGs. This finding could be consistent with the notion that L-DOPA treatment in the dopamine-depleted forebrain increases excitatory transmission from the thalamus to cortical areas but reduces excitatory inputs from the cortex to the striatum (De Deurwaerdère et al., 2017). As previously indicated, the L-DOPA dose used in the study by Smith and colleagues (2016) was considerably lower (4 mg/kg), which may also contribute to the observed differences. Furthermore, we note that the previous proteomic and transcriptomic analyses performed on samples taken postmortem from PD patients indicated neurodegeneration-related gene expression in the prefrontal cortex (Dumitriu et al., 2016). Conversely, there was no indication of frontal cortex degeneration after 6-OHDA lesioning in rats (Simola et al., 2007), which may partially explain the lack of differences in gene expression between hemispheres that we observed. Finally, 6-OHDA lesioning causes the loss of dopaminergic neurons within days of treatment, whereas degeneration develops over years before the onset of motor impairments in PD patients (Morrish et al., 1998). Thus, although there is overlap to some extent in gene expression changes reported here and in previous studies on postmortem cortex samples, e.g., *Zfp189*, *Klhl21*, *Bcl6*, and *Dddit4* (Dumitriu et al., 2016) or *Nab2* and *Sox2* (Dumitriu et al., 2012), a direct comparison of 6-OHDA lesioning with postmortem analyses does not appear valid. We consider this to be the primary limitation of our study.

Our data showed that L-DOPA treatment had a robust effect on gene expression in the frontal cortex. The effects of L-DOPA appeared to involve two components: a general response and a drug-specific response. Both would be of relevance in the context of the mechanisms through which L-DOPA treatment affects cognitive functions requiring cortical activity. We note extensive similarities between changes in gene expression induced by L-DOPA treatment and those induced by other drugs, but we cannot exclude the possibility that these changes are specific to selected cell types. Thus, the specific aspect of drug-induced gene expression could be the profile of the cells affected rather than the exact list of affected transcripts. Independently, as shown in the results, a single induced transcript, *Grasp* (general receptor for phosphoinositides 1 associated scaffold protein, also known as *Tamalin*), appeared to be specific to the effects of L-DOPA. Caution should be taken in the case of such singular results, but we note that the Grasp protein is involved in the trafficking of metabotropic glutamate receptors (Kitano et al., 2002). In addition, its increased abundance was reported in postmortem CA1 hippocampal region samples from persons diagnosed with schizophrenia (Matosin et al., 2015). *Grasp* is upregulated in the ipsilateral striatum of rats with 6-OHDA unilateral lesions following chronic L-DOPA exposure as well as in Flinders-resistant line rats that developed abnormal involuntary movements (a symptom of L-DOPA-induced dyskinesia; Schintu et al., 2020). These observations support the notion that *Grasp* could be part of a mechanism involved in the effects of L-DOPA on cognitive functions; however, this notion remains a conjecture.

An additional point to consider is the cellular selectivity of the response to L-DOPA. The method employed here to determine the cell-type specificity of gene expression does not provide direct information on cell-level gene expression changes; rather, we extrapolate data based on the fraction of neuronal and nonneuronal cells that were observed to express the genes of interest. The analysis suggests a heterogeneous response to L-DOPA in the frontal cortex involving different subtypes of neurons, glia, macrophages and vascular cells. This finding is also consistent with previous reports assessing the functions of the differentially expressed genes. In the case of *Sgk1*, which has extensively annotated isoforms, the change in expression was selective for specific transcripts with the canonical, astroglia-enriched and neuron-enriched variant being upregulated and neuron-specific downregulated (Arteaga et al., 2008; Slezak et al., 2013). Similarly, *Slc2a1*, which encodes the GLUT1 glucose transporter, is considered a marker of the cells forming the blood-brain barrier, whereas neurons predominantly express GLUT3 (Xiuli et al., 2005). Taken together, these results show that the observed changes in the transcriptome may also affect nonneuronal cells.

A major caveat of analysis based on whole-tissue RNA-Seq is that the changes we observe occur exclusively in subsets of the cells indicated and speculatively may even involve lesion-induced or L-DOPA-induced transcription in cells that normally do not express the differentially regulated genes.

Taken together, the results of gene expression analysis in the context of previously reported data lead to two conclusions. First, the effects of L-DOPA on the frontal cortex are mainly not lateralized and thus substantially differ from reported transcription changes in the striatum. Second, the genes affected by L-DOPA overlap with those reported in the cases of several other drugs; hence, the key feature associated with the clinical effects of L-DOPA is probably cell-type and transcript-isoform specificity. We believe that extending the analysis of the effects of L-DOPA on gene expression to achieve both cell type and anatomic resolution could lead to the identification of patterns associated with antiparkinsonian efficacy.

## Synthesis

Reviewing Editor: Alfonso Araque, University of Minnesota Decisions are customarily a result of the Reviewing Editor and the peer reviewers coming together and discussing their recommendations until a consensus is reached. When revisions are invited, a fact-based synthesis statement explaining their decision and outlining what is needed to prepare a revision will be listed below. The following reviewer(s) agreed to reveal their identity: Angela Cenci.

The reviewers have found merit and interest on the findings reported. I concur with the reviewers’ comments expressing the interest of the study. However, they also expressed some concerns about several issues that need to be addressed and clarified. I also consider pertinent the concerns expressed.

Reviewers and I believe that the comments require less than two months of work.

I am looking forward to receiving a revised version of the manuscript with the reviewers´ comments addressed.

Alfonso Araque

Reviewing Editor

eNeuro

Comments of the reviewers:

Reviewer 1.

In this study, L-DOPA treatment was given to rats with unilateral lesions of dopaminergic neurons, and the frontal cortex was processed for RNAseq analysis. The results show bilateral changes in gene expression in L-DOPA-treated animals vs. saline-treated controls. The differentially expressed transcripts are functionally diverse and include many genes involved in the regulation of transcription. A bioinformatic assessment of the cell-type specificity of gene expression changes indicated that these occurred in both neuronal and non-neuronal cell types. A comparison with published databases indicated that a large part of the differentially expressed genes overlapped with genes regulated by other drugs.

Being the first L-DOPA-transcriptome study focusing on the frontal cortex, the study is novel and timely, and the bioinformatics analyses appear to have been well-performed. There are, however, some significant limitations that the authors will need to discuss and justify:

1) Methods, L-DOPA treatment: the chosen dose (12,5 mg/kg/day i.p.) is rather high for MFB-lesioned animals. Why was this dose chosen, and how many of the treated rats developed dyskinesia during the treatment?

2) Methods, apomorphine-induced rotation: explain why that particular cut-off value of rotations was chosen and what level of dopamine depletion this corresponds too (and measured how...).

3) “Frontal cortex samples from both hemispheres were dissected...”: what cortical areas were actually included in the tissue sample (e.g. motor, premotor, prelimbic, cingulate, etc) ? Please give this important information with reference to well-known rat brain atlases (e.g. by Paxinos).

4) “The 12,455 genes were clustered with the “Dynamic Tree Cut” method, which

resulted in the detection of 17 modules, each labeled with a color (Figure 3A). There were 5 modules detected at the merging threshold of 0.5 (Figure 3-1): “brown"-7148 genes (including 6909 protein coding genes), “cyan"-1450 genes (1401), “gray"-287 genes (257), “midnight blue"-3490 genes (3388) and “salmon"-80 genes (79)”.

- Please explain the significance of the colours assigned by this bioinformatic analysis, and also explain why precisely these colours were chosen (does the colour carry any particular information?).

5) Related to the above point, the authors’ frequent referring to the so-called “salmon module” throughout the manuscript sounds really confusing because the term is devoid of any biological significance per se. Author should clearly define what the salmon colour representation is supposed to indicate (or, what is the common denominator of gene changes included in this category).

6) “The observed changes may not necessarily be primarily related to neurons..:” this statement (which is placed in the Results!) needed to be substantiated with data. The authors should moreover justify why they did not try to validate claims of cellular localization by immunohistochemistry (or in situ hybridization) on a separate set of animals.

7) Discussion: “Thus, either the unilateral lesion had no appreciable basal effect on homeostasis in the frontal cortex, or the effects on both sides were the same”. An alternative and most likely explanation is that changes occurring in unilaterally affected functional areas (e.g., M1, see Halje et al PMID: 23175810) were diluted because the large tissue samples used in this study mainly consisted of areas that had been affected non-specifically by the drug treatment.

Reviewer 2.

In this manuscript, the authors use RNA sequencing analysis to investigate whether and how L-dopa treatment alters gene expression in the frontal cortex in parkinsonian rats. As the authors point out, Parkinson’s Disease has a variety of symptoms, including cognitive symptoms, some of which may derive from frontal cortical dysfunction, and the origins of these symptoms at the molecular, cellular, and circuit level has been little studied. The current study parallels prior work that has been done in several laboratories that focused on gene expression changes in the striatum in rodent models of Parkinson’s Disease, with and without treatment with L-dopa.

For these reasons, studies on cortical function in animal models of Parkinson’s Disease are especially valuable. Changes in frontal cortical gene expression patterns would inform future studies that examine cellular dysfunction or circuit-level changes in vivo. The manuscript is well-organized and clearly written. The authors find 48 genes that are differentially regulated in the L-dopa-treated condition, which are similar on the left and right hemispheres (despite being a unilateral depletion model). They go on to examine the 48 genes through weighted gene coexpression analysis, and find many are in a small gene module. They also look within the gene expression data for changes by cell type, and generally find that none of the 48 genes was exclusive to one of the canonical cell type examined. Finally, they compare the changes in gene expression to those seen with other pharmacological agents (including some that would not be predicted to be related to levodopa structurally or in mechanisms of action), and find substantial overlap, without many distinctly regulated genes.

Major comments:

1. The main results can be construed as mostly “negative” in nature - there are not surprising or cell-type specific changes in gene expression in the cortex, and in fact changes are bilaterally expressed. To make these findings accessible and interesting to a broad audience of investigators interested in the cellular/molecular mechanisms of Parkinson’s Disease and L-dopa treatment, it would be useful to lead readers through the Results section with more explicit predictions/hypotheses, and the rationale for each type and level of analysis they employ. This could be just an additional sentence at the beginning of the section for each type of analysis, but would greatly improve the readability to a broad audience.

2. Interpretation of the main results depend on the efficacy of the dopamine depletion, and the authors state these brain samples were taken from mice in another study. However, likely given the author-blind review, it is hard for me to assess the validation of these mice (postmortem staining for tyrosine hydroxylase, motor behavior in the parkinsonian and L-dopa treated state).

3. It is hard to interpret these results without some tissue validation (eg with in situ hybridization or immunohistochemistry) of the genes cited, as is commonly done in other gene expression studies.

4. The authors frequently invoke cortical cell types in both the Results and discussion sections. In fact, there are some cortical neurons that express dopamine receptors, and these would be candidate neurons to show more marked changes in gene expression with L-dopa (as is true in the striatum). But as far as I can tell this specific hypothesis was not tested. It would be very useful, if their data has adequate statistical power, to include testing of specific hypotheses.

5. It appears that there are 5 rats/group included in this study? Given the variability in the 6-OHDA rodent model of Parkinson’s Disease, this seems like a low number. Can the authors more explicitly describe the variability from one animal to another, and likewise if they were able to make any comparisons between gene expression and behavioral results (as it sounds as if these animals were tested for behavioral parameters in another study). However, if the N is truly this small, this is likely not possible.

## Author Response

Comments of the reviewers:

Reviewer 1.

In this study, L-DOPA treatment was given to rats with unilateral lesions of dopaminergic neurons, and the frontal cortex was processed for RNAseq analysis. The results show bilateral changes in gene expression in L-DOPA-treated animals vs. saline-treated controls. The differentially expressed transcripts are functionally diverse and include many genes involved in the regulation of transcription. A bioinformatic assessment of the cell-type specificity of gene expression changes indicated that these occurred in both neuronal and non-neuronal cell types. A comparison with published databases indicated that a large part of the differentially expressed genes overlapped with genes regulated by other drugs.

Being the first L-DOPA-transcriptome study focusing on the frontal cortex, the study is novel and timely, and the bioinformatics analyses appear to have been well-performed. There are, however, some significant limitations that the authors will need to discuss and justify:

1) Methods, L-DOPA treatment: the chosen dose (12,5 mg/kg/day i.p.) is rather high for MFB-lesioned animals. Why was this dose chosen, and how many of the treated rats developed dyskinesia during the treatment?

A high dose was selected to observe robust L-DOPA effects on gene expression. The specific dose was selected based on the upper range reported in the literature in rats with unilateral lesions in MFB e.g. (Conti et al., 2016; Fieblinger et al., 2018; Gerlach, Beck, Riederer, & Van Den Buuse, 2011; Gerlach, Van Den Buuse, Blaha, Bremen, & Riederer, 2004; Smith, Hardy, Schallert, & Lee, 2016; Xie et al., 2014). Testing for dyskinesia was not performed.

2) Methods, apomorphine-induced rotation: explain why that particular cut-off value of rotations was chosen and what level of dopamine depletion this corresponds too (and measured how...).

The cut-off value of 98 was used in a previous study based on the exactly the same method. In this study, all samples were actually derived from rats that performed > 120 rotations. In the revised version of the manuscript we added a supplementary table with number of contra- and ipsiversive rotations for each animal (Figure 1-1), and we also modified Figure 1 by adding plots showing decreases in tissue dopamine levels in the striatum on the lesioned side for each of the rat groups.

3) “Frontal cortex samples from both hemispheres were dissected...”: what cortical areas were actually included in the tissue sample (e.g. motor, premotor, prelimbic, cingulate, etc) ? Please give this important information with reference to well-known rat brain atlases (e.g. by Paxinos).

The samples included: orbitofrontal, limbic, cingulate, primary and secondary motor, primary somatosensory and insular cortex (Bregma 4.2 mm to 2.2 mm based on (Paxinos & Watson, 1986). The description of the cortical areas dissected was added in the Materials and Methods section of the paper accordingly.

4) “The 12,455 genes were clustered with the “Dynamic Tree Cut” method, which

resulted in the detection of 17 modules, each labeled with a color (Figure 3A). There were 5 modules detected at the merging threshold of 0.5 (Figure 3-1): “brown"-7148 genes (including 6909 protein coding genes), “cyan"-1450 genes (1401), “gray"-287 genes (257), “midnight blue"-3490 genes (3388) and “salmon"-80 genes (79)”.

- Please explain the significance of the colours assigned by this bioinformatic analysis, and also explain why precisely these colours were chosen (does the colour carry any particular information?).

The algorithm of the WGCNA package assigns each gene cluster a color. Referring to gene clusters by color names is a commonly used convention in papers where the WGCNA method is used (e.g., Pei, Chen, & Zhang, 2017; Saad et al., 2019; Santpere et al., 2018; Wendeln et al., 2018). The colors themselves have no inherent meanings. We have clarified this under Materials and Methods and Results sections.

5) Related to the above point, the authors’ frequent referring to the so-called “salmon module” throughout the manuscript sounds really confusing because the term is devoid of any biological significance per se. Author should clearly define what the salmon colour representation is supposed to indicate (or, what is the common denominator of gene changes included in this category).

As noted by the Reviewer, the term ‘salmon’ has no inherent meaning, and is used to denote a group of genes comprising one of the identified modules. We have revised the Materials and Methods and Results sections to note this explicitly. We revised the manuscript text to clarify this matter.

6) “The observed changes may not necessarily be primarily related to neurons..:” this statement (which is placed in the Results!) needed to be substantiated with data.

We agree that the statement was too speculative to be placed under the Results section and thus was removed. The Discussion section have been also modified, and this part concludes with a much more balanced tone.

The authors should moreover justify why they did not try to validate claims of cellular localization by immunohistochemistry (or in situ hybridization) on a separate set of animals.

Again, we agree that proper validation of cell-type specificity of expression would require an independent experiment confirming co-localization of specific transcripts and/or proteins with cell-type markers. This is beyond the scope of this report, and we toned down the associated statements on cell-type specificity under Results and Discussion.

However, we still want to emphasize this point since it may be crucial in the context of analyses of gene ontology terms. Potential differences in cell-type specificity of transcripts regulated by L-DOPA implies that they are unlikely to belong to a single network, rather they belong to separate network depending on cell-type affected. Moreover, in the case of similarities with transcription signatures associated with other drugs, if the changes potentially occur in glial or endothelial cells rather than neurons, the extensive similarities with drugs acting primarily on peripheral tissues become less surprising.

7) Discussion: “Thus, either the unilateral lesion had no appreciable basal effect on homeostasis in the frontal cortex, or the effects on both sides were the same”. An alternative and most likely explanation is that changes occurring in unilaterally affected functional areas (e.g., M1, see Halje et al PMID: 23175810) were diluted because the large tissue samples used in this study mainly consisted of areas that had been affected non-specifically by the drug treatment.

The M1 cortex was reported to be involved in the motor effects of dopamine depletion and L-DOPA-induced dyskinesia. We agree that a unilateral effect in the M1 cortex could had been diluted in the case of the experiments described in the manuscript. However, please note that the highest expression of dopamine receptors in the rodent cortex is found in the orbitofrontal, prefrontal and cingulate cortices, and we had assumed that effects associated with the side of lesion should be the strongest there. Additionally, a report based on experiments with similar design showed no clear lateralization of lesion effects in the cortex (Monnot, Zhang, Nikkhou-Aski, Damberg, & Svenningsson, 2017). Monnot and colleagues specifically showed that Fos expression was affected bilaterally across the cortex areas examined, even though they appeared lateralized in the striatum. This is in our opinion consistent with results originally reported by Berke and colleagues (Berke, Paletzki, Aronson, Hyman, & Gerfen, 1998).

We have updated the Discussion section to make note of the possibility that localized lateralized differences in gene expression could be lost due to the relatively large part of the frontal cortex being assessed.

Reviewer 2.

In this manuscript, the authors use RNA sequencing analysis to investigate whether and how L-dopa treatment alters gene expression in the frontal cortex in parkinsonian rats. As the authors point out, Parkinson’s Disease has a variety of symptoms, including cognitive symptoms, some of which may derive from frontal cortical dysfunction, and the origins of these symptoms at the molecular, cellular, and circuit level has been little studied. The current study parallels prior work that has been done in several laboratories that focused on gene expression changes in the striatum in rodent models of Parkinson’s Disease, with and without treatment with L-dopa.

For these reasons, studies on cortical function in animal models of Parkinson’s Disease are especially valuable. Changes in frontal cortical gene expression patterns would inform future studies that examine cellular dysfunction or circuit-level changes in vivo. The manuscript is well-organized and clearly written. The authors find 48 genes that are differentially regulated in the L-dopa-treated condition, which are similar on the left and right hemispheres (despite being a unilateral depletion model). They go on to examine the 48 genes through weighted gene coexpression analysis, and find many are in a small gene module. They also look within the gene expression data for changes by cell type, and generally find that none of the 48 genes was exclusive to one of the canonical cell type examined. Finally, they compare the changes in gene expression to those seen with other pharmacological agents (including some that would not be predicted to be related to levodopa structurally or in mechanisms of action), and find substantial overlap, without many distinctly regulated genes.

Major comments:

1. The main results can be construed as mostly “negative” in nature - there are not surprising or cell-type specific changes in gene expression in the cortex, and in fact changes are bilaterally expressed. To make these findings accessible and interesting to a broad audience of investigators interested in the cellular/molecular mechanisms of Parkinson’s Disease and L-dopa treatment, it would be useful to lead readers through the Results section with more explicit predictions/hypotheses, and the rationale for each type and level of analysis they employ. This could be just an additional sentence at the beginning of the section for each type of analysis, but would greatly improve the readability to a broad audience.

The results were revised accordingly. Each paragraph was updated to include a statement of purpose.

2. Interpretation of the main results depend on the efficacy of the dopamine depletion, and the authors state these brain samples were taken from mice in another study. However, likely given the author-blind review, it is hard for me to assess the validation of these mice (postmortem staining for tyrosine hydroxylase, motor behavior in the parkinsonian and L-dopa treated state).

In the revised version of the manuscript we added supplementary table with number of contra- and ipsiversive rotations for each animal (Figure 1-1), and we also modified Figure 1 by adding plots showing decreases in tissue dopamine levels in the striatum on the lesioned side for each of the rat groups. As presented on Figure B and C, mean dopamine tissue content has decreased by more than 99% in the left striatum as compared to the right striatum in both groups.

3. It is hard to interpret these results without some tissue validation (eg with in situ hybridization or immunohistochemistry) of the genes cited, as is commonly done in other gene expression studies.

As noted in the response to Reviewer 1, we believe that validation of the tissue specificity is not as straightforward as suggested by the reviewer (if we understand the criticism correctly). First difficulty is that the method would have to be validated for each target transcript/protein (i.e. specificity and sensitivity). Second, it would be necessary to co-stain with a cell type marker, or preferably, use a genetically modified animals expressing fluorescent proteins in specific cell types. With regard to the latter, few such models are available in the case of rats. Finally, in some cases there is already available data confirming bilateral L-DOPA induced-expression after unilateral, lesion i.e. (Berke et al., 1998; Monnot et al., 2017). In the revised manuscript we have updated the discussion, further exploring cell-type expression specificity of Slc2a1 and Sgk1 (the latter on the transcript isoform level) and toning down our conclusions.

4. The authors frequently invoke cortical cell types in both the Results and discussion sections. In fact, there are some cortical neurons that express dopamine receptors, and these would be candidate neurons to show more marked changes in gene expression with L-dopa (as is true in the striatum). But as far as I can tell this specific hypothesis was not tested. It would be very useful, if their data has adequate statistical power, to include testing of specific hypotheses.

We agree that the statements about cell type expression specificity were too speculative to be placed under Results and thus removed. The corresponding part of Discussion section has also been changed.

Again, we agree that proper validation of cell-type specificity of expression would require an independent experiment confirming co-localization of specific transcripts and/or proteins with cell-type markers or use of different sequencing method. This is beyond the scope of this report, thus we toned down the associated statements on cell-type specificity under Results and Discussion.

However, we still want to emphasize cell-type expression specificity since it may be crucial in the context of analyses of gene ontology terms. Potential differences in cell-type specificity of transcripts regulated by L-DOPA implies that they are unlikely to belong to a single network, rather they belong to separate network depending on cell-type affected. Moreover, in the case of similarities with transcription signatures associated with other drugs, if the changes potentially occur in glial or endothelial cells rather than neurons, the extensive similarities with drugs acting primarily on peripheral tissues become less surprising.

5. It appears that there are 5 rats/group included in this study? Given the variability in the 6-OHDA rodent model of Parkinson’s Disease, this seems like a low number. Can the authors more explicitly describe the variability from one animal to another,

The 98 cut-off was used to select animals in the cohort that was previously reported and was used in this study to assess gene expression. In fact, all samples analyzed in this study were derived from rats that performed > 120 rotations. A table listing numbers of rotations for each animal is now included as supplementary material (Figure 1-1).

While we agree that variability in unilateral 6-OHDA-induced model is a confounding factor, please note that the design of the analysis compares the lesioned and unlesioned sides in a pairwise manner (mixed model ANOVA was used). This approach was selected specifically to limit the effect of potential differences in the extent of the lesion. This point was emphasized in the revised Results (second paragraph).

Finally, the results from gene expression analysis pass significance criteria that fully account for the number of comparisons performed. We cannot exclude the possibility that some moderate differences between the lesioned and unlesioned sides were not detected.

and likewise if they were able to make any comparisons between gene expression and behavioral results (as it sounds as if these animals were tested for behavioral parameters in another study).

There were no statistically significant correlations between the number of rotations (the only behavioral type of test performed on these animals) and the levels of the differentially expressed genes.

However, if the N is truly this small, this is likely not possible.

We agree that the size of the sample is low for robust regression analysis, however, the number of samples analyzed is arguably typical for RNA-seq based experiments and does not deviate from the literature (e.g., Duan et al., 2018; Song et al., 2018).

Bibliography:

Berke, J. D., Paletzki, R. F., Aronson, G. J., Hyman, S. E., & Gerfen, C. R. (1998). A complex program of striatal gene expression induced by dopaminergic stimulation. The Journal of Neuroscience : The Official Journal of the Society for Neuroscience, 18(14), 5301-5310.

Conti, M. M., Meadows, S. M., Melikhov-Sosin, M., Lindenbach, D., Hallmark, J., Werner, D. F., & Bishop, C. (2016). Monoamine transporter contributions to L-DOPA effects in hemi-parkinsonian rats. Neuropharmacology, 110, 125-134.

Duan, X., Gan, J., Xu, F., Li, L., Han, L., Peng, C., ... Peng, D. (2018). RNA sequencing for gene expression profiles in a rat model of middle cerebral artery occlusion. BioMed Research International, 2018.

Fieblinger, T., Zanetti, L., Sebastianutto, I., Breger, L. S., Quintino, L., Lockowandt, M., ... Cenci, M. A. (2018). Striatonigral neurons divide into two distinct morphological-physiological phenotypes after chronic L-DOPA treatment in parkinsonian rats. Scientific Reports, 8(1), 10068.

Gerlach, M., Beck, J., Riederer, P., & Van Den Buuse, M. (2011). Flibanserin attenuates L-DOPA-sensitized contraversive circling in the unilaterally 6-hydroxydopamine-lesioned rat model of Parkinson’s disease. Journal of Neural Transmission, 118(12), 1727-1732.

Gerlach, M., Van Den Buuse, M., Blaha, C., Bremen, D., & Riederer, P. (2004). Entacapone increases and prolongs the central effects of L-DOPA in the 6-hydroxydopamine-lesioned rat. Naunyn-Schmiedeberg’s Archives of Pharmacology, 370(5), 388-394.

Monnot, C., Zhang, X., Nikkhou-Aski, S., Damberg, P., & Svenningsson, P. (2017). Asymmetric dopaminergic degeneration and levodopa alter functional corticostriatal connectivity bilaterally in experimental parkinsonism. Experimental Neurology, 292, 11-20.

Paxinos, G., & Watson, C. (1986). The Rat Brain in Stereotaxic Coordinates (2nd ed.). Academic Press.

Pei, G., Chen, L., & Zhang, W. (2017). WGCNA Application to Proteomic and Metabolomic Data Analysis. Methods in Enzymology (1st ed., Vol. 585). Elsevier Inc.

Saad, M. H., Rumschlag, M., Guerra, M. H., Savonen, C. L., Jaster, A. M., Olson, P. D., ... Bannon, M. J. (2019). Differentially expressed gene networks, biomarkers, long noncoding RNAs, and shared responses with cocaine identified in the midbrains of human opioid abusers. Scientific Reports, 9(1), 1-9.

Santpere, G., Garcia-Esparcia, P., Andres-Benito, P., Lorente-Galdos, B., Navarro, A., & Ferrer, I. (2018). Transcriptional network analysis in frontal cortex in Lewy body diseases with focus on dementia with Lewy bodies. Brain Pathology, 28(3), 315-333.

Smith, E. S., Hardy, G. A., Schallert, T., & Lee, H. J. (2016). The impact of L-dopa on attentional impairments in a rat model of Parkinson’s disease. Neuroscience, 337, 295-305.

Song, S. H., Jang, W. J., Hwang, J., Park, B., Jang, J. H., Seo, Y. H., ... Jeong, C. H. (2018). Transcriptome profiling of whisker follicles in methamphetamine self-administered rats. Scientific Reports, 8(1), 1-12.

Wendeln, A.-C., Degenhardt, K., Kaurani, L., Gertig, M., Ulas, T., Jain, G., ... Neher, J. J. (2018). Innate immune memory in the brain shapes neurological disease hallmarks. Nature, 556(7701), 332-338.

Xie, C. L., Wang, W. W., Zhang, S. F., Yuan, M. L., Che, J. Y., Gan, J., ... Liu, Z. G. (2014). Levodopa/benserazide microsphere (LBM) prevents L-dopa induced dyskinesia by inactivation of the DR1/PKA/P-tau pathway in 6-OHDA-lesioned Parkinson’s rats. Scientific Reports, 4, 1-10.
